# Evaluation of Antidepressant Prescribing in Treatment-Resistant Depression: A Clinical Audit on Adherence to NICE Guidelines and Its Impact on Patient Outcomes

**DOI:** 10.1192/bjo.2025.10639

**Published:** 2025-06-20

**Authors:** Sarah Mostafa, Aiden Hargreaves, Micheal Kurkar

**Affiliations:** Pennine Care NHS Trust, Greater Manchester, United Kingdom

## Abstract

**Aims:** Treatment-Resistant Depression (TRD) is diagnosed when patients fail to respond to at least two adequate trials of antidepressant medication. Patients with TRD are often referred for Transcranial Magnetic Stimulation (TMS) as a next-line treatment. However, delays in recognizing TRD and inappropriate medication management may prolong suffering and consume unnecessary resources.

Hypothesis: Patients with TRD experience prolonged delays in receiving effective treatment due to non-adherence to NICE guidelines, leading to extended suffering and increased healthcare resource consumption.

**Methods:** A retrospective audit was conducted on 50 patients referred for TMS with a confirmed diagnosis of TRD. Patients were classified as meeting criteria for TRD if they had received at least two different antidepressants at an appropriate dose for a minimum of four weeks during the current depressive episode. We analysed whether medication management adhered to the National Institute for Health and Care Excellence (NICE) guidelines for depression treatment.

**Results:** Among the 50 patients, 70% didn’t follow NICE guidelines for depression management, with a significant proportion remaining on ineffective antidepressant regimens beyond the recommended duration. This resulted in delays in initiating alternative treatments, prolonging the duration of depressive episodes, and leading to unnecessary resource consumption.

Additionally, the mean duration between the first and second antidepressant was found to be 46 days (approximately 1.5 months), while the mean duration between the second antidepressant and the initiation of TMS was approximately 751 days (almost 2 years). Furthermore, 21 out of 50 patients (42%) had comorbid psychiatric disorders alongside depression.

**Conclusion:** Both primary and secondary care patients remained on antidepressants for a prolonged duration beyond NICE recommendations. This delay increased the time patients struggled with depression without receiving effective treatment. The prolonged ineffective medication use led to unnecessary consumption of healthcare resources. Delays in adjusting treatment plans postponed further treatment approaches, such as augmentation strategies or referral for TMS.

To optimize TRD management, early identification and adherence to NICE guidelines are essential. Regular reviews help discontinue ineffective treatments and ensure timely referrals to TMS. Enhanced clinical training and integrated mental health pathways improve treatment access and outcomes.

